# Poor Clinical Outcomes among Pneumonia Patients with Depressive Disorder

**DOI:** 10.1371/journal.pone.0116436

**Published:** 2014-12-31

**Authors:** Li-Ting Kao, Shih-Ping Liu, Herng-Ching Lin, Hsin-Chien Lee, Ming-Chieh Tsai, Shiu-Dong Chung

**Affiliations:** 1 Graduate Institute of Life Science, National Defense Medical Center, Taipei, Taiwan; 2 Sleep Research Center, Taipei Medical University Hospital, Taipei, Taiwan; 3 Department of Urology, National Taiwan University Hospital and College of Medicine, Taipei, Taiwan; 4 School of Health Care Administration, Taipei Medical University, Taipei, Taiwan; 5 Department of Psychiatry, School of Medicine, Taipei Medical University, Taipei, Taiwan; 6 Division of Gastroenterology, Department of Internal Medicine, Cathay General Hospital, Taipei, Taiwan; 7 Division of Urology, Department of Surgery, Far Eastern Memorial Hospital, Banciao, Taipei, Taiwan; University of Dundee, United Kingdom

## Abstract

**Background:**

Some studies suggested that psychological stress may be associated with the severity and duration of infectious diseases. In this population-based study, we investigated associations between depressive disorder (DD) and pneumonia outcomes in Taiwan with a large-scale database from the National Health Insurance.

**Methods:**

Our study defined 112,198 patients who were hospitalized with a principal diagnosis of pneumonia. We defined their admission date for treatment of pneumonia as the index date. Subsequently, we selected 2,394 patients with DD within 3 years prior to their index date and 11,970 matched patients without DD. We carried out separate conditional logistic regressions to explore the association of clinical pneumonia treatment outcome (ICU admission, use of mechanical ventilation, acute respiratory failure and in-hospital death) with previously diagnosed DD.

**Results:**

Patients with DD had a significantly higher probability of an intensive care unit admission (18.1% vs. 12.9%; *p*<0.001), need for mechanical ventilation (21.9% vs. 18.1%; *p*<0.001) and in-hospital death (10.4% vs. 9.0%; *p* = 0.025) than patients without DD. The study showed that pneumonia patients with DD were respectively 1.41- (95% CI: 1.25∼1.59, *p*<0.001), 1.28- (95% CI: 1.14∼1.43, *p*<0.001), and 1.17- times (95% CI: 1.01∼1.36, *p* = 0.039) greater odds of being admitted to the ICU, need for mechanical ventilation, and in-hospital death than patients without DD after adjusting for monthly income, urbanization level, geographic region and Charlson Comorbidity Index score.

**Conclusions:**

In conclusion, we found that pneumonia patients with DD were associated with poor treatment outcomes compared to patients without DD.

## Introduction

Pneumonia is a major cause of hospitalization and is frequently associated with great morbidity, mortality, and utilization of healthcare resources [1], [2]. In the US, pneumonia annually affects more than four million adults and accounts for more than one million hospital admissions [3]. Recently, some studies attempted to explore the relationship between pneumonia and chronic diseases [4]–[6]. Moreover, a study in the United States has found that the hospitalization for pneumonia increased the risk of subsequent depression, functional disability and cognitive impairment [7]. However, few studies have explored the association between mental disorders and subsequent pneumonia outcomes [8].

Depressive disorder (DD) is a widespread chronic disease which is associated with substantial mortality, comorbidities, and disabilities [9]–[12]. Numerous studies reported that depression and psychological stress can induce dysfunction of the immune system and modulate the production of proinflammatory cytokines [13]–[15]. These changes in the human body are large enough to be clinically significant [14]. For example, one recent population-based study reported that depression was associated with subsequent hospitalization for pneumonia [16]. In addition, some studies found that depression may contribute to delayed wound healing and increase complication risks after an injury [17], [18].

Although various studies found a relationship among depression, infectious diseases, and associated immunological mechanisms, very few studies have specifically investigated outcomes of hospitalization due to pneumonia among subjects diagnosed with DD by physicians. One previous study found that the use of antidepressants or benzodiazepines was related to a severe prognosis of total community-acquired lower respiratory tract infections (LRTIs) in the elderly aged ≥60 years [19]. However, it still remains unclear about detailed treatment outcomes for hospitalizations due to pneumonia in adults with and those without DD.

This population-based study hypothesized that the pneumonia patients with DD might have poor clinical outcomes in comparison with the patients without DD. The aim of this study was to investigate disparate clinical outcomes (i.e., intensive care unit (ICU) admission, use of mechanical ventilation, acute respiratory failure, and in-hospital death) of pneumonia patients with or those without DD.

## Methods

### Database

Data for this population-based retrospective study were taken from the Taiwan Longitudinal Health Insurance Database 2000 (LHID2000). This study was exempt from full review by the Institutional Review Board of National Defense Medical Center because the LHID2000 consists of de-identified secondary data released to the public for research purposes. Taiwan's National Health Insurance (NHI) program, which was initiated in 1995, provides comprehensive and affordable medical care for all its citizens. The LHID2000 contains claims data of 1,000,000 individuals randomly selected from the 2000 Registry of Beneficiaries (*n* = 23.72 million) of the Taiwan NHI program. The LHID2000 enables researchers to trace all medical services of these 1,000,000 enrollees since the beginning of Taiwan's NHI program. The LHID2000, which was open to the researchers in Taiwan, was available from the NHRI (http://nhird.nhri.org.tw/date_01.html). High validity of data derived from the Taiwanese NHI program was demonstrated by many researchers and the Taiwan National Health Research Institute [20], [21].

### Study Sample

We first identified 112,198 patients who were hospitalized with a principal diagnosis of pneumonia (ICD-9-CM 480∼483.8, 485∼487.0) from January 2005 to December 2011. We selected only the first episode for inclusion in this study (*n* = 59,736) if a patient had more than one admission for treatment of pneumonia during the study period. We then excluded patients under 18 years of age (*n* = 13,385) in order to limit the study to the adult population. As a result, 46,351 pneumonia patients met our selection criteria. We defined their admission date for treatment of pneumonia as the index date. Of the selected pneumonia patients, 2,860 had received a diagnosis of DD (ICD-9-CM codes 296.2, 296.3, 300.4, and 311) within 3 years prior to their index date. A 3-year period was chosen to examine the association because DD tended to be chronic with the median time to remission being close to one year in previous studies, Furthermore, we also excluded patients who were diagnosed with other psychiatric disorders (*n* = 466) such as bipolar disorder (ICD-9-CM codes 296.1), schizophrenia (ICD-9-CM codes 295) and anxiety disorders (ICD-9-CM codes 309.81∼309.83, 300.01, 300.02, 300.1, and 300.2) because these disorders have been found to be associated with worse clinical outcomes among subjects hospitalized for medical-surgical conditions. Finally, we assigned 2,394 pneumonia patients with DD as the study group herein.

We further extracted a comparison group from the remaining 43,491 pneumonia patients. We randomly selected 11,970 pneumonia patients (five for each patient with DD) matched with the study group in terms of gender, age (18∼24, 25∼34, 35∼44, 45∼54, 55∼64, 65∼74 and ≧75 years), and the year of the index date using the SAS (SAS, Cary NC, USA) proc surveyselect program. We also ensured that none of the selected comparison patients had any medical records of DD or other psychiatric disorders such as bipolar disorder, schizophrenia and anxiety disorders since the initiation of the NHI program in 1995. Therefore, we could not rule out the possibility that the selected study patients may have had DD or other psychiatric disorders prior to 1995.

### Variables of Interest

The primary study outcomes included ‘ICU admission’, ‘use of mechanical ventilation’, ‘acute respiratory failure’, and ‘in-hospital death’. They were all binary variables. In addition, ‘in-hospital death’ was defined as ‘the death of a patient at any time after admission if the patient did not leave the hospital’.

### Statistical Analysis

All analyses were conducted using the SAS system. We used Chi-squared tests to compare differences in patients' monthly income, geographic location (northern, central, eastern, and southern Taiwan), urbanization level (5 levels, with 1 the most and 5 the least urbanized), and Charlson comorbidity index (CCI) score between cases and controls. The CCI was used to quantify preexisting comorbidities as a means of adjusting for the higher mortality risks associated with 19 medical conditions (congestive heart failure, myocardial infarction, liver disease, cancer, dementia, etc.).

We carried out separate conditional logistic regressions (stratified by gender, age group, index year, and hospital) to explore the association of clinical pneumonia treatment outcome with previously diagnosed DD. A *p* value of <0.05 was used to assess statistical significance in this study.

## Results

Of the 14,364-person study sample, the mean age was 68.8 years with a standard deviation of 16.9 years. Demographic characteristics and CCI scores of the study and comparison groups are presented in Table 1. After matching for gender, age group, and the year of the index date, no significant difference was observed in CCI scores between patients with and those without DD. However, there were significant differences in monthly income (*p*<0.001) and geographic region (*p* = 0.006) between patients with and those without DD.

**Table 1:** Demographic characteristics of pneumonia patients with and those without depressive disorder (DD) in Taiwan in 2005∼2011 (*n* = 14,364).

Variable	Patients with DD (*n* = 2,394)		Comparison group (*n* = 11,970)		*p* value
	Total no.	Percent (%)	Total No.	Percent (%)	
Gender					1.000
Male	1,274	53.2	6,370	53.2	
Female	1,120	46.8	5,600	46.8	
Age (years)					1.000
18∼24	36	1.5	180	1.5	
25∼34	85	3.6	425	3.6	
35∼44	144	6.0	720	6.0	
45∼54	206	8.6	1,030	8.6	
55∼64	314	13.1	1,570	13.1	
65∼74	471	19.7	2,355	19.7	
≧75	1,138	47.5	5,690	47.5	
Monthly income (US$)					<0.001
$1∼530	1,532	64.0	6,988	58.4	
$530∼830	690	28.8	4,028	33.7	
≥$830	172	7.2	954	8.0	
Urbanization level					0.053
1 (most urbanized)	594	24.8	2,786	23.3	
2	631	26.4	2,942	24.6	
3	349	14.6	1,797	15.0	
4	402	16.8	2,168	18.1	
5 (least urbanized)	418	17.5	2,277	19.0	
Geographic region					0.006
Northern	978	40.9	4,886	40.8	
Central	543	22.7	3,010	25.2	
Southern	795	33.2	3,618	30.3	
Eastern	78	3.3	456	3.8	
Charlson Comorbidity Index score					0.254
0	911	38.1	4,544	38.0	
1	749	31.3	3,777	31.6	
2	328	13.7	1,483	12.4	
3	195	8.2	977	8.2	
≥4	211	8.8	1,189	9.9	

Table 2 show the distribution of treatment outcomes between pneumonia patients with and those without DD. Patients with DD had significantly higher probabilities of ICU admission (18.1% vs. 12.9%; *p*<0.001), need for mechanical ventilation (21.9% vs. 18.1%; *p*<0.001) and in-hospital death (10.4% vs. 9.0%; *p* = 0.025) than patients without DD. However, there was no significantly difference in the outcome of acute respiratory failure.

**Table 2:** Frequencies and proportions of adverse clinical outcomes for pneumonia patients with or those without depressive disorder (DD) in Taiwan.

Presence of adverse clinical outcomes	Total sample (*n* = 14,364)	Subject with DD (*n* = 2,394)	Comparison group (*n* = 11,970)
	n (%)	n (%)	n (%)
Intensive care unit admission	1,976 (13.8)	434 (18.1)	1,542 (12.9)
Acute respiratory failure	2,732 (19.0)	481 (20.1)	2,251 (18.8)
Need for mechanical ventilation	2,690 (18.7)	524 (21.9)	2,166 (18.1)
In-hospital death	1,326 (9.2)	250 (10.4)	1,076 (9.0)

Table 3 presents the crude odds ratios (ORs) and 95 confidence intervals (CIs) of ICU admission, acute respiratory failure, the need for mechanical ventilation, and in-hospital death between pneumonia patients with and those without DD. Separate conditional logistic regressions (stratified by gender, age group, index year,and hospital) revealed that patients with DD were 1.43- (95% CI: 1.26∼1.61, *p*<0.001), 1.30- (95% CI: 1.16∼1.45, *p*<0.001), and 1.18- times (95% CI: 1.02∼1.37, *p* = 0.025) greater odds of being admitted to the ICU, need for mechanical ventilation, and in-hospital death compared to patients without DD. Table 3 further shows adjusted ORs of ICU admission, acute respiratory failure, the use of mechanical ventilation, and in-hospital death between pneumonia patients with and those without DD. Conditional logistic regression analyses (stratified by gender, age group, index year, and hospital) suggested that patients with DD were 1.41- (95% CI: 1.25∼1.59, *p*<0.001), 1.28- (95% CI: 1.14∼1.43, *p*<0.001), and 1.17- times (95% CI: 1.01∼1.36, *p* = 0.039) greater odds of being admitted to the ICU, need for mechanical ventilation, and in-hospital death than patients without DD after adjusting for monthly income, urbanization level, geographic region and Charlson Comorbidity Index score.

**Table 3:** Crude and adjusted odds ratios (ORs) and 95% confidence intervals (CIs) for adverse clinical outcomes for pneumonia patients with or those without depressive disorder (DD) in Taiwan.

Presence of adverse clinical outcomes	Subject with DD (*n* = 2,394)	Comparison group (*n* = 11,970)
Intensive care unit admission		
OR (95% CI)	1.43*** (1.26∼1.61)	1.00
Adjusted OR (95% CI)	1.41*** (1.25∼1.59)	1.00
Acute respiratory failure		
OR (95% CI)	1.09 (0.98∼1.23)	1.00
Adjusted OR (95% CI)	1.08 (0.96∼1.21)	1.00
Need for mechanical ventilation		
OR (95% CI)	1.30*** (1.16∼1.45)	1.00
Adjusted OR (95% CI)	1.28*** (1.14∼1.43)	1.00
In-hospital death		
OR (95% CI)	1.18* (1.02∼1.37)	1.00
Adjusted OR (95% CI)	1.17* (1.01∼1.36)	1.00

Note:

* *p*<0.05;

** *p*<0.01;

*** *p*<0.001;

The adjusted OR was calculated by a conditional logistic regression which was adjusted for monthly income, urbanization level, geographic region and Charlson Comorbidity Index score.

## Discussion

To date, numerous studies have reported the relationship between depression and pneumonia. For example, one study in the United States has concluded that the hospitalization for pneumonia increased the risk of subsequent depression (OR: 1.63; 95% CI: 1.06∼2.51) and moderate-to-severe cognitive impairment (OR: 2.46; 95% CI: 1.60∼3.79) [7]. On the other hand, a recent population-based study found that depression was independently associated with 1.28-times greater odds of hospitalization for pneumonia in comparison to those without depression. This relationship persisted after adjusting for baseline cognitive and functional status (OR: 1.24; 95% CI: 1.03∼1.50) [16]. Both studies have consistently established the relationship between depression and pneumonia. The present study further assessed treatment outcomes for pneumonia between patients with or those without DD. We found that pneumonia patients with DD had higher odds of poor clinical outcomes, including ICU admission, the need for mechanical ventilation, and in-hospital death compared to pneumonia patients without DD.

Aggravated treatment outcomes in infected patients with depression were reported in other studies. They found that psychological stress or depression was associated with infectious diseases, and also affected wound healing and complication risks [16]–[18], [22]–[25]. Moreover, some studies indicated that depression and psychological stress may induce an immune function imbalance and stimulate the production of proinflammatory cytokines, such as interleukin (IL)-1, IL-6 and tumor necrosis factor (TNF) [13]–[15]. Additionally, levels of those proinflammatory cytokines were associated with disease degeneration [26], [27]. One meta-analysis revealed that an elevated level of inflammatory markers might reduce the lung function of patients with COPD [26]. Another study also showed that a higher circulating concentration of IL-6 indicated exacerbation of a hemodynamic condition and increasing heart failure symptoms in patients with congestive heart failure [27]. In addition, Yende et al. found a relationship between higher levels of baseline TNF and IL-6 in the systemic circulation of elderly individuals and an increased risk of subsequently developing community-acquired pneumonia (CAP) requiring hospitalization [28]. They reported that the highest tertiles of TNF and IL-6 were independent predictors of CAP susceptibility, and the adjusted ORs were 1.6 (95% CI: 1.0∼2.7) and 1.7 (95% CI: 1.1∼2.8). Consequently, increasing levels of proinflammatory cytokines in patients with DD might potentially be the reason for the poor pneumonia treatment outcomes in our study.

In addition, the negative attitude of patients to receiving medical therapy, such as worse adherence for chronic medical conditions and delayed treatment-seeking might be potential factors which affected pneumonia outcome in subjects with DD. A meta-analysis study reported that the odds were 3 times (95% CI: 1.96∼4.89) higher that depressed subjects would be noncompliant with medical treatment recommendations than non-depressed subjects [29]. Moreover, other studies have revealed that patients with mental disorders could delay help-seeking behavior, reducing the likelihood of detection and diagnosis [30]. Therefore, subjects with DD may be predisposed to worse disease outcomes when confronted with acute infection such as pneumonia.

A specific strength of our study is the use of a population-based dataset in Taiwan. This feature afforded sufficient statistical power and an adequate sample size to detect differences in pneumonia treatment outcomes between the two groups after adjusting for confounders.

Nevertheless, there are several limitations of this study. First, the NHIRD database which we used in this study did not provide lifestyle, health information or the disease severity of subjects, such as the intensity of tobacco use, drug abuse, alcohol consumption, body-mass index or the CURB-65 Pneumonia Severity Score which might possibly affect the relationship between DD and treatment outcomes for respiratory diseases. Second, this database might not entirely represent patients with DD. Some subjects with DD might not have been searching for healthcare services or have received medication therapy for psychiatric disorders, because they thought that associated treatments were not necessary or the disease was embarrassing. Therefore, ICD-9-CM codes which were diagnosed by physicians might not include some subjects with mild to moderate DD. The potential effects of DD might be under-estimated in the study. Third, our study lacked the data about timing of the receiving of antibiotics for pneumonia. It may be one of the plausible factors which explained acute respiratory failure secondary to pneumonia and/or pneumonia-related mortality. Finally, most of the patients included in our study were of Chinese ethnicity, so the ability to generalize the results to other ethnic groups is not certain.

Our population-based study found that pneumonia patients with DD were associated with an increased risk of poor treatment outcomes, including ICU admission, the need for mechanical ventilation and in-hospital death compared to pneumonia patients without DD. We suggest that medical professionals and policymakers should provide suitable mental health care to the subjects with acute infection by focusing on existing healthcare programs and health policy. It might be ultimately beneficial in improving clinical outcomes of the pneumonia for subjects with DD.
